# Epiduroscopic decompression of a symptomatic perineural cyst: A case report

**DOI:** 10.1097/MD.0000000000017564

**Published:** 2019-11-01

**Authors:** Sangmin Jeong, Francis Sahngun Nahm, Jae-Sung Lee, Woong Ki Han, Eunjoo Choi, Pyung-Bok Lee, Ho-Jin Lee

**Affiliations:** a Department of Anesthesiology and Pain Medicine, Seoul National University Bundang Hospital, Seongnam; b Department of Anesthesiology and Pain Medicine, College of Medicine, Seoul National University; c Department of Anesthesiology and Pain Medicine, Seoul National University Hospital, Seoul, South Korea.

**Keywords:** back pain, decompression, pain clinics, spinal canal, tarlov cyst

## Abstract

**Rationale:**

Perineural cysts in the spinal canal are usually asymptomatic. However, symptoms can occur when the cyst becomes large enough to compress a nerve root. There are still no established treatment options for this disease. In this report, we describe a case of successful decompression of the large symptomatic perineural cyst using epiduroscope.

**Patient concerns:**

A 42-year-old male patient visited our pain center complaining of discomfort and pain in his right posterior thigh. Magnetic resonance imaging of the patient showed a large perineural cyst (53 × 31 × 21 mm) compressing the right S1 nerve. No other abnormalities that would explain the patient's symptoms were identified.

**Diagnosis:**

Perineural cyst at the right S1 nerve.

**Interventions:**

We performed an epiduroscopic decompression of the perineural cyst. After advancing the epiduroscope and locating the cyst, we used the laser to make a hole in the cyst wall. Then, the epiduroscope was advanced into the cyst, and the cystic fluid was aspirated.

**Outcomes:**

The symptoms of the patient were relieved after the procedure, without any complications. There was no recurrence of symptoms until 6 months after the procedure.

**Lessons:**

The epiduroscope is a minimally invasive method which can be used safely for decompression of symptomatic perineural cysts in the spinal canal.

## 1 Introduction

Perineural cysts, also called Tarlov cysts, were first described by Dr Tarlov in 1938.^[1]^ These cysts are usually incidental findings on magnetic resonance imaging (MRI) and are commonly observed in the sacral spine. They are usually asymptomatic; however, in some cases, they can cause symptoms, especially when they are large enough to compresses the nerve root. So far, various treatments methods have been utilized, as there is no standard treatment for symptomatic lumbosacral perineural cysts. After excluding other causes of the patient's symptoms, conservative treatment (e.g., physiotherapy and oral medications) is implemented first.^[2]^ If the symptoms persist despite these treatments, cyst decompression is considered.^[3]^ There are surgical and non-surgical methods for decompression of a cyst. With the recent trend towards minimally invasive procedures, a variety of non-surgical methods are being attempted.^[4,5]^ In this paper, we report a successful case of decompression of a symptomatic perineural cyst in the sacral canal using an epiduroscope. The patient gave the informed consent to publish this case. This case report was approved by the Institutional Review Board of the Seoul National University Bundang Hospital (IRB No. B-1809-495-701).

## 2 Case report

**Figure 1:**
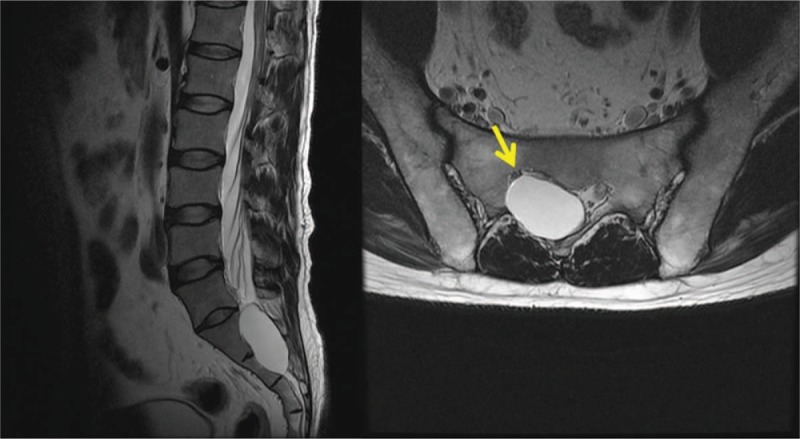
Magnetic resonance imaging of the patient complaining of low back pain and right posterior thigh pain. A large perineural cyst is observed (size: 53 × 31 × 21 mm). The arrow indicates the compressed right S1 nerve root.

**Figure 2:**
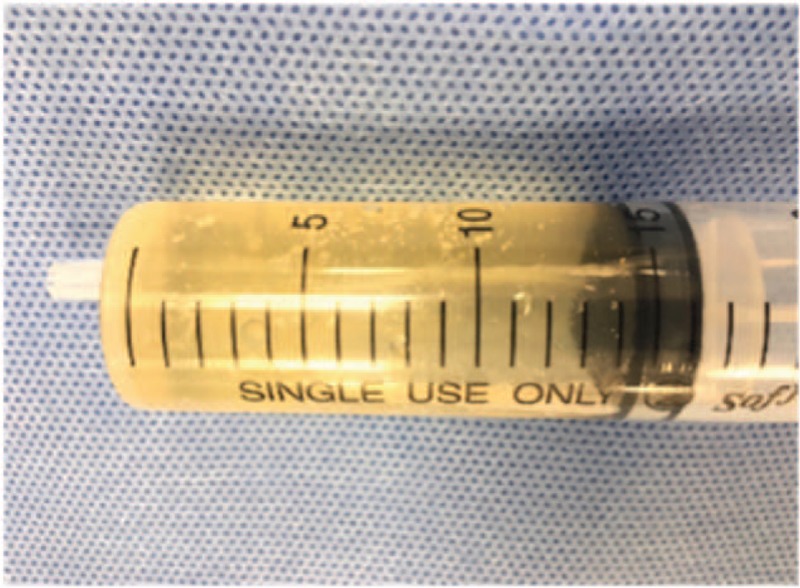
The aspirated fluid. It was yellowish and slightly sticky like apple juice.

**Figure 3:**
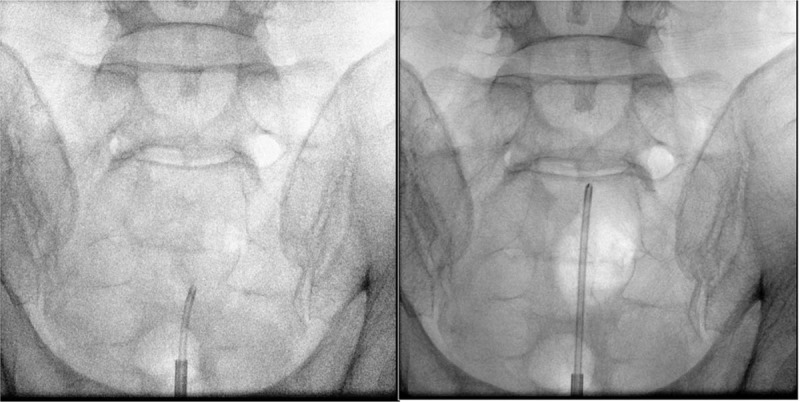
Fluoroscopic image of the procedure. The epiduroscope is inserted through the sacral canal.

A 42-year-old male patient visited our pain center complaining of discomfort in the right posterior thigh, which started a year ago. He reported that prolonged standing or sitting worsened his symptoms (numeric rating score 8, 0; no pain, 10 maximum pain imaginable), and driving for more than an hour was difficult because of the pain. The patient's pain eased when he lay down. Over the past 7 months, the patient had received manipulation therapy and physiotherapy at other hospitals, along with the following oral medications: aceclofenac 100 mg 1 tab bid, limaprost 5 mcg 1 tab bid, and eupatilin 60 mg 1 tab bid; however, the treatments were not effective. On physical examination, there were no abnormalities identified in the lower back or lower extremity, and motor and sensory testing were normal. On the lumbosacral spine MRI obtained at an external hospital, a cystic lesion was observed on the right side of the midline, ranging from S1 to S2 (size: 53 mm × 31 mm × 21 mm). The lesion showed low signal intensity on the T1-weighted image and high signal intensity on the T2-weighted image; it was located in the path of the right S1 nerve root and was compressing the nerve (Fig. 1). No other abnormal findings that could explain the patient's symptoms were observed on the MRI. Therefore, a transforaminal injection was performed with a mixture of 3 ml of 0.18% ropivacaine and 5 mg of dexamethasone. Three months later, the patient reported that the symptoms had not improved. We again tried to aspirate the cyst via the caudal and right S1 transforaminal approach, but we were unsuccessful. Therefore, we inserted a needle through the L5-S1 interlaminar space and were able to partially aspirate the cystic fluid (approximately 15 mL). The aspirated fluid was yellowish and slightly sticky like apple juice (Fig. 2). Two weeks later, at a follow-up visit, the patient stated that the aspiration had improved his symptoms, but it was still difficult to stand or drive for long periods. Therefore, we planned to perform an epiduroscopic cyst decompression through a caudal approach for a more complete decompression of the cyst. For the epiduroscopic decompression, the patient lay prone, with vital sign monitoring and an intravenous infusion of normal saline. Aseptic skin preparation was performed with betadine soap and 2% chlorohexidine; then, a sterile surgical drape was applied. To allow for insertion of the large bore cannula, local anesthesia was performed with 1% lidocaine 3 cm distal to the sacral hiatus. Then, a working cannula was inserted through the sacral hiatus under fluoroscopic guidance. An epiduroscopic device (2010 standard video guided catheter, Myelotec Inc, GA) and a Holmium:Yag laser (VeraPulse P200, Lumenis Ltd., Israel) were inserted though the cannula. Using fluoroscopic guidance, the position of the epiduroscope was frequently checked as it was advanced to the S2 level, where the cyst wall was identified (Fig. 3).

**Figure 4:**
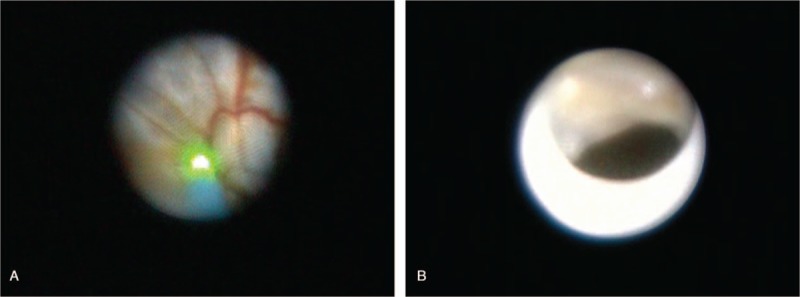
(A) Epiduroscopic view of the cyst wall. The green color indicates the Holmium:Yag laser targeting the cyst wall. (B) A hole (arrow) made by the laser is observed.

**Figure 5:**
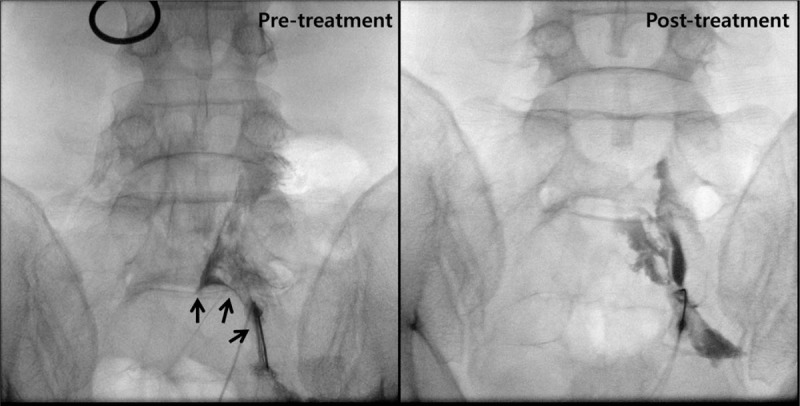
The pre/post-treatment fluoroscopic images. The cyst that had compressed the right S1 nerve root was decompressed after the epiduroscopic procedure. The arrows indicate the cyst wall.

We were able to compress the cyst wall with the epiduroscopic device, but were unable to puncture the cyst wall with the mechanical pressure. Therefore, we made a hole in the cyst wall using the Holmium:Yag laser and inserted the epiduroscope inside the cyst (Fig. 4). The fluid inside the cyst was aspirated as completely as possible (total 35 mL) and submitted to the laboratory for cell analysis. Four holes were made in the cyst wall with the laser, in order to complete the decompression and to prevent re-filling. Radio-opaque dye was injected through a needle inserted into the right S1 foramen to check whether the cyst was decompressed. The cyst that had compressed the right S1 nerve root was decompressed after the epiduroscopic procedure. The pattern of dispersion of the contrast medium observed along the path of S1 nerve after cyst decompression was considerably different from that observed before cyst decompression (Fig. 5). After the procedure, the patient was discharged from the hospital that day, without any complications, such as post-dural puncture headache. On the follow up visit one week after the procedure, the patient stated that the discomfort and pain in the right posterior thigh disappeared and that he could drive for a long period without pain. There was no recurrence of symptoms until six months after the procedure. The cytological evaluation revealed that the cystic fluid contained only red blood cells (37,500/mL), without other cells, such as white blood cells or malignant cells.

## 3 Discussion

In this case, symptoms caused by nerve root compression from a large perineural cyst in the sacral canal were successfully relieved using epiduroscopic decompression. Perineural cysts occur in 1.5% of the total population, and approximately 13% of cases are symptomatic.^[6]^ On histopathological examination of eight specimens, nerve fibers were present in 75%, ganglion cells in 25%, and evidence of previous hemorrhage in 50%.^[7]^ When the cerebrospinal fluid (CSF) fills and inflates a cyst, it causes nerve compression, which can induce perineal pain, low back pain, and sciatica.^[8]^ Although the exact mechanism is unknown, recent studies have shown that the unidirectional check valve mechanism could be the main cause of the cyst's size increase.^[5]^ For the patient in this case, there was a cystic lesion in the posterior-superior part of the nerve pathway. Therefore, maintaining a standing or sitting position for a long time caused pain by increasing the pressure of the cyst on the nerves, and lying down relieved the pain by decreasing the pressure. The cyst was easily diagnosed on MRI, showing low signal intensity on the T1-weighted image and high signal intensity on the T2-weighted image.^[6,9]^ Symptomatic therapy is commonly preferred for perineural cysts, but surgical and non-surgical methods of cyst removal are considered in cases with non-adaptive pain.^[3]^ Current surgical methods used are laminectomy for cyst decompression and nerve root resection; laminoplasty and cyst fenestration; incision and drainage; and lumbo-peritoneal CSF shunting. Open surgery allows for visualization of the entire lesion, but the wide and invasive approach required presents additional risks, such as infection, bleeding, nerve damage, and leakage of the CSF.^[10,11]^ Commonly executed non-surgical methods are computed tomography (CT)-guided cyst rupture, fluoroscopic fenestration and injection, and trans-foraminal approach and cyst aspiration. It has been reported that relapse is common if anatomical diversity prevents access to the lesion or there is communication with the CSF.^[11]^ The development of endoscopy equipment has led to a variety of treatments for epidural lesions, which are considered more effective compared with the existing CT or fluoroscopy-guided procedures. In this case, we first attempted fluoroscopy-guided decompression; however, since the aspiration of the cyst was not complete, and the symptoms persisted, we performed epiduroscopic decompression. It has been reported that surgical removal of multiple small cysts and endoscopic removal of a single, large cyst in the central region had excellent results.^[5]^ In the patient in this report, a relatively large single cyst that occurred in the central part was thought to be old enough to have caused bone deformation. The color of the fluid aspirated from the cyst was yellowish and sticky like apple juice and the small reduction in the size of the cyst improved the patient's symptoms. Therefore, endoscopic removal was planned, since little to no communication with the CSF was suspected. In cases that are clinically ambiguous or when a more objective evaluation is required, myelography can be performed to assess the inside of the cyst and evaluate for potential communication with the CSF.^[12]^ Moreover, if communication is identified, the area can be accessed using an endoscope, and a laser can be used to cauterize the area or block the entrance with materials, such as fibrin glue.^[13]^ However, there has been a report of meningitis that occurred after treatment using fibrin glue.^[10]^ In addition to perineural cysts, it is also possible to remove lesions inside the spinal canal, such as facet cysts^[14]^ and discal cysts,^[15]^ using a flexible scope. Using the flexible scope, multiple lesions can be removed simultaneously; therefore, the application range of endoscopic procedures will likely continue to widen in the future. The limitations of this case were the absence of cyst fluid analysis prior to epiduroscopic decompression and the lack of histologic examination of the cyst wall for exact classification of the cyst.

In conclusion, this case demonstrates that the epiduroscope can be used for decompression of symptomatic perineural cysts in the spinal canal, especially when the cyst is large enough to cause symptoms.

## Author contributions

**Conceptualization:** Francis Sahngun Nahm.

**Project administration:** Francis Sahngun Nahm.

**Supervision:** Francis Sahngun Nahm.

**Validation:** Eunjoo Choi, Pyung-Bok Lee.

**Visualization:** Jae-Sung Lee.

**Writing – original draft:** Sangmin Jeong.

**Writing – review & editing:** Francis Sahngun Nahm, Woong Ki Han, Ho-Jin Lee.

Francis Sahngun Nahm orcid: 0000-0002-5900-7851.
